# Supplementary material for: Single Cell RNA-Seq Identifies Immune-Related Prognostic Model and Key Signature-SPP1 in Pancreatic Ductal Adenocarcinoma
Source: Genes (Basel). 2022 Sep 29;13(10):1760. doi: 10.3390/genes13101760 (PMC9601640; doi:10.3390/genes13101760)
Supplement: Supplementary file 1 [file genes-13-01760-s001.zip › genes-1899367-supplementary.pdf]

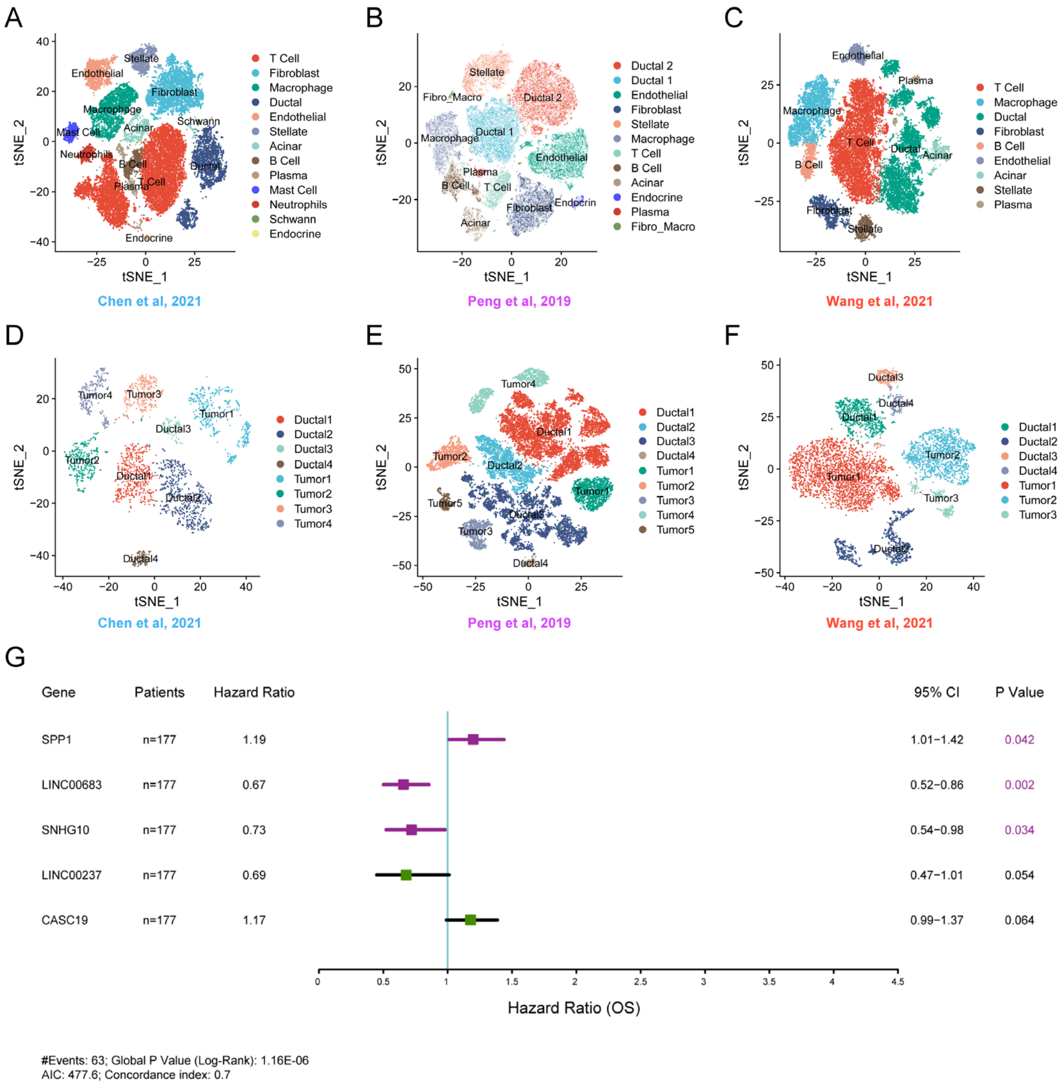


**Figure S1. scRNA-seq analysis identified cell subpopulations of PDAC.** (A-F) The t-SNE plots showing the original cluster (A-C) and named cell subpopulations (D-F) for multiple human scRNA-seq datasets; (G) The forest plot showing the prognostic values of signatures in established prognostic model.

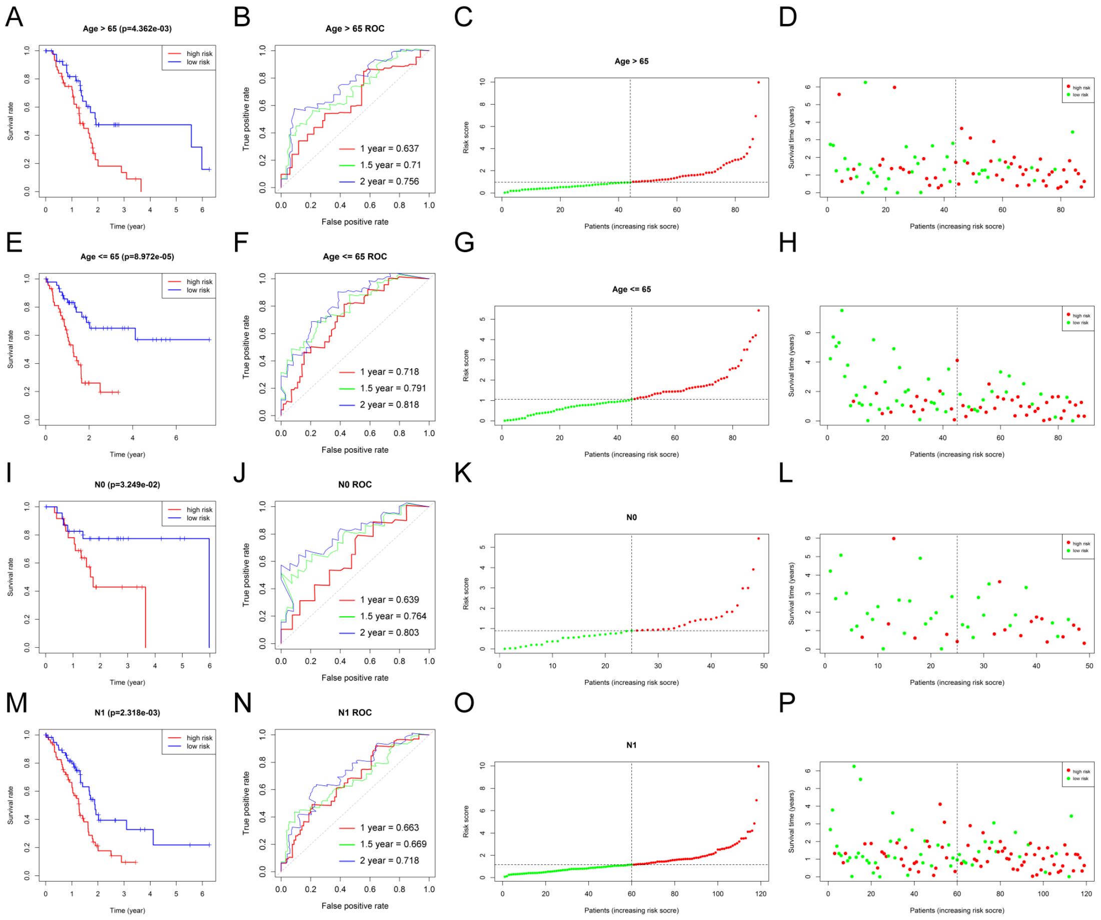


**Figure S2. The performance of established prognostic model in different age and N stage subgroups.** (A-H) The performance of prognostic model in different age subgroups, KM curve showing different OSs between high and low risk groups (A, E), ROC curve was used to evaluate the accuracy of prognostic model for 1-/1.5-/2-year OSs (B, F), risk score distribution of subjects in train set (C, G) and survival status scatter plot (D, H); (I-P) The performance of prognostic model in different N stage subgroups.

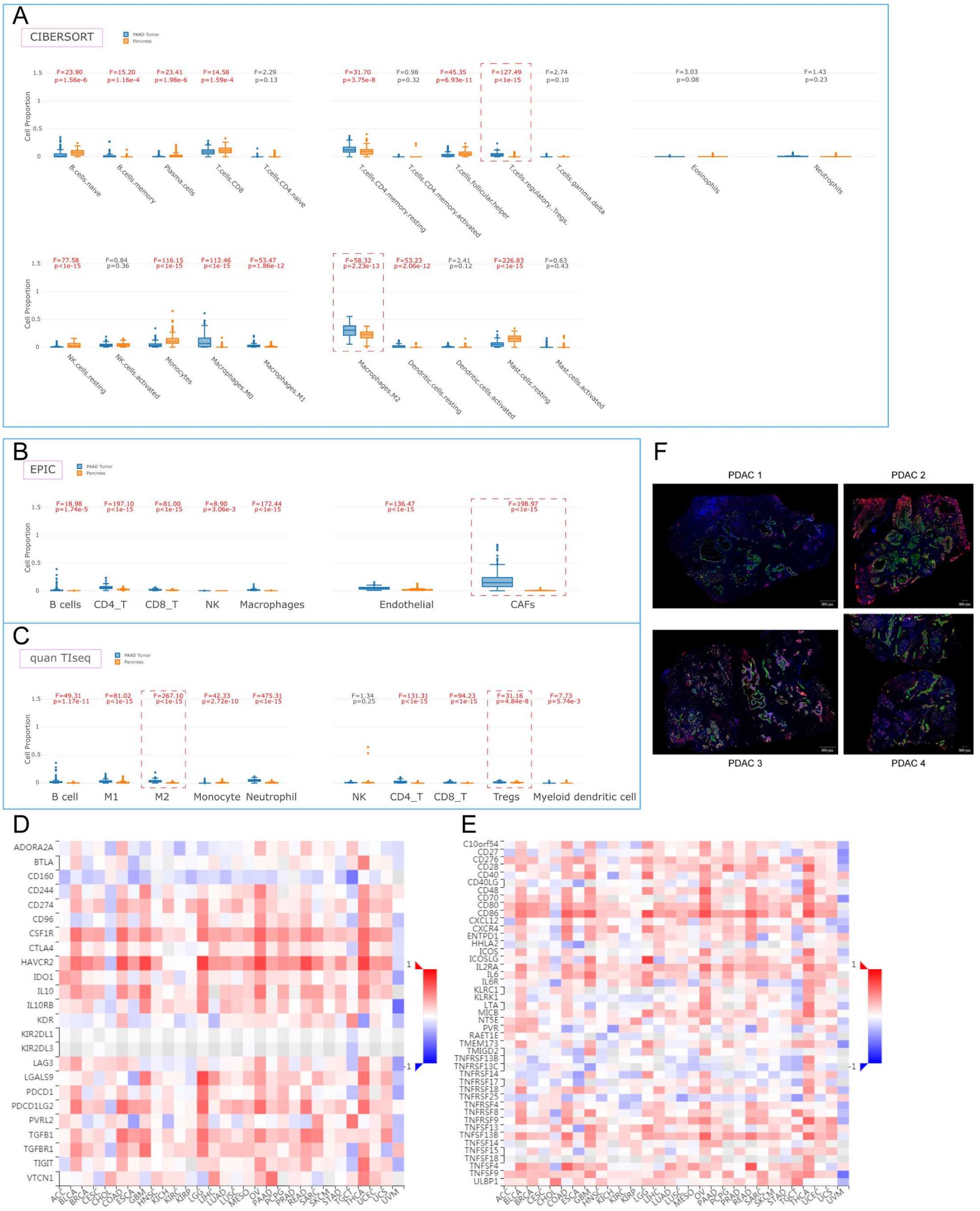

**Figure S3. The cell compositions of PDAC and normal pancreatic tissue based on deconvolution analyses.** (A-C) Multiple deconvolution-based bioinformatics tools were used to compare the cell compositions between PDAC and normal pancreatic tissues; (D-E) The heatmap showing the correlation between the expression of SPP1 and immunoinhibitors (D) and immunostimulators (E); (F) The panorama of multicolor immunohistochemistry for PDAC tissues. Red and blue represent positive and negative correlation, respectively. The intensity of color indicates correlation index (from -1 to 1).
